# Adherence and weight loss with a total diet replacement programme for increased cancer risk associated with obesity: a UK cohort study

**DOI:** 10.1136/bmjopen-2026-119498

**Published:** 2026-09-28

**Authors:** Helen Clarke, Cheryl Lombardelli, Emma Barrett, Julie Wisely, Katharine Sellers, Basil Issa, Anthony Howell, Emma J Davidson, Sacha Howell, Michelle Harvie

**Affiliations:** 1 Department of Gynaecological Oncology, St. Mary’s Hospital, Manchester University NHS Foundation Trust, Manchester, UK; 2 Division of Cancer Sciences, The University of Manchester, Manchester, UK; 3 The Prevent Breast Cancer Research Unit, Manchester University NHS Foundation Trust, Manchester, UK; 4 Research and Innovation, Manchester University NHS Foundation Trust, Manchester, UK; 5 Greater Manchester Mental Health NHS Foundation Trust, Manchester, UK; 6 Department of Endocrinology and Diabetes, Manchester University NHS Foundation Trust, Manchester, UK; 7 Manchester Academic Health Science Centre, Manchester, UK

**Keywords:** Obesity, NUTRITION & DIETETICS, Primary Prevention

## Abstract

**Objective:**

To evaluate engagement and weight loss with a 12-month total diet replacement (TDR) programme in women with increased risk of breast and endometrial cancer, living with obesity.

**Design:**

Prospective cohort study.

**Setting:**

Secondary care.

**Participants:**

47 premenopausal women living with obesity.

**Intervention:**

12-month programme including a 12-week TDR phase with biopsies of the breast and endometrium, before and after, followed by 4 weeks of diet reintroduction and a weight maintenance/continued weight loss phase (weeks 17–52). Primary and secondary outcome measures: This paper reports retention and adherence to the programme, weight loss, changes in mental well-being, quality of life (QoL), weight loss self-efficacy, physical activity and fidelity of delivery of the programme.

**Results:**

37 women (79%) completed the whole programme. Completers and those who withdrew, respectively, had a mean (SD) age of 41.4 years (5.7) vs 38.1 (5.8) and baseline body mass index of 36.2 (4.9) kg/m^2^ vs 40.0 (7.9) kg/m^2^ and 76% vs 90% had children 18 or younger living at home. Median (IQR) weight was reduced at all time points; −10.8 kg (−13.8, −7.2) at 3 months, −11.5 kg (−15, −7.6) at 6 months, −11.0 kg (−16.4, −7.8) at 9 months and −8.4 kg (−13.9, −3.7) at 12 months. Across the 12-month programme, the median (IQR) contacts with the study dietitians per participant was 28 (25, 33) and median contact hours were 7.6 (6.6, 10.6). 68% received psychological support. QOL improved; OWL-QOL (Obesity and Weight Loss)−17 (−24.8, −7.8) and EQ5D 1 (0, 2), with reductions in binge eating scores −4 (−6.5, −0.8) but no changes in mental health or physical activity scales.

**Conclusions:**

TDR could be a weight loss programme for women at increased risk of breast and endometrial cancers related to obesity. Future controlled trials with extended follow-up are required before recommending routine implementation of TDR in high-risk clinics.

**Trial registration number:**

ISRCTN15358157.

**STRENGTHS AND LIMITATIONS OF THIS STUDY**

- Evaluation of a total diet replacement (TDR) weight loss programme in women at increased risk of two obesity-related cancers.
- Remotely delivered TDR weight loss programme.
- There was no control group that did not receive the TDR programme.
- The cohort included selected and potentially highly motivated people recruited to a breast and endometrial biopsy study.
- The cohort included selected and potentially highly motivated people recruited to a breast and endometrial biopsy study.

## Introduction

 Obesity and overweight are strong and potentially modifiable risk factors for developing breast and endometrial cancer. Maintained intentional weight loss of greater than or equal to 5% has been associated with reduced risk of developing postmenopausal breast cancer.^1^ Recent biomarker studies suggest even greater reductions in the risk of breast and other obesity-related cancers with weight loss of at least 10%.^2 3^ Bariatric surgery has been shown to achieve an average weight loss between 20% and 30% and reduce the risk of breast and endometrial cancer by ~50%.^4–7^ However, it is not acceptable to many patients nor feasible as a roll-out cancer prevention strategy in the UK owing to the economic burden.^8 9^ Low energy total diet replacement (TDR) weight loss programmes, that is, 12 weeks of ~850 kcal (3556 kJ), have been shown to induce at least 5% and 10% wt loss in 73% and 45%, respectively, of individuals with obesity within primary care.^10^ TDR programmes are estimated to be cost-effective in this setting^11^ and could be a viable weight loss intervention for reducing cancer risk in those at increased risk. However, within this specific population, there is no data on adherence, retention, psychological outcomes or efficiency. This paper summarises findings from a cohort study using a multidisciplinary TDR weight loss programme among women living with obesity and increased risk of breast and/or endometrial cancer. We report programme adherence, weight loss, changes in diet and physical activity health behaviours, mental well-being, quality of life (QoL), weight loss self-efficacy and fidelity of delivery of the programme.

## Methods

### Study design

PRevention Of Breast and Endometrial cancer using TDR was a prospective, non-blinded, randomised controlled trial among 47 women with obesity at increased risk of breast and/or endometrial cancer. Randomisation was 2:1 to either an immediate 12-month TDR weight loss programme (n=30) or a delayed 12-month TDR weight loss programme (control) who maintained their normal diet for 3 months before starting the programme (n=17). The primary aim of the study was to assess cellular and molecular changes in breast and endometrial tissue following 3-months TDR-induced weight loss versus 3 months of normal diet in the control group, which will be reported elsewhere. This cohort study reports secondary outcomes (adherence, changes in well-being and eating behaviours scores, in addition to fidelity of delivery of the programme) as the RCT was powered for reduction in tissue biomarkers. The control group is valid for the first 3-month analysis then both groups receive the full TDR intervention programme.

The inclusion criteria were age 30–50 years, body mass index (BMI) ≥30 kg/m^2^ or ≥27.5 kg/m^2^ if from a high-risk minority ethnic group, premenopausal with reported regular menstrual cycles and willing to use non-hormonal contraception (barrier or abstinence) during the TDR phase of the study. Exclusion criteria included previous breast or endometrial cancer, hormonal contraceptive use within the preceding 3 months, tamoxifen or anti-progestin use in the preceding 6 months, previous bariatric surgery, currently pregnant or breast feeding, prescribed prohibited medications, that is, anticoagulants, antipsychotics, weight loss medications and certain mental health diagnoses as identified through medical history or baseline screening questionnaires (online supplemental table 1). Patients with existing type 2 diabetes mellitus (T2DM) were eligible for inclusion, however, we excluded those found to have potential new diagnosis of T2DM on screening bloods.

Participants were primarily recruited from a high-risk breast cancer risk prediction and prevention clinic and internal advertisements to staff at the Manchester University Hospitals NHS Foundation Trust and University of Manchester.

### Patient and public involvement

Prior to ethical submission, we conducted a focus group including four premenopausal women, not associated with the study or study team, at increased risk of breast to discuss the acceptability of an interventional prevention study including tissue biopsies. A second group of premenopausal women reviewed the patient information sheet and consent form.

### The 12-month TDR programme

The TDR programme is described within the protocol publication^12^ and in figure 1.

**Figure 1:**
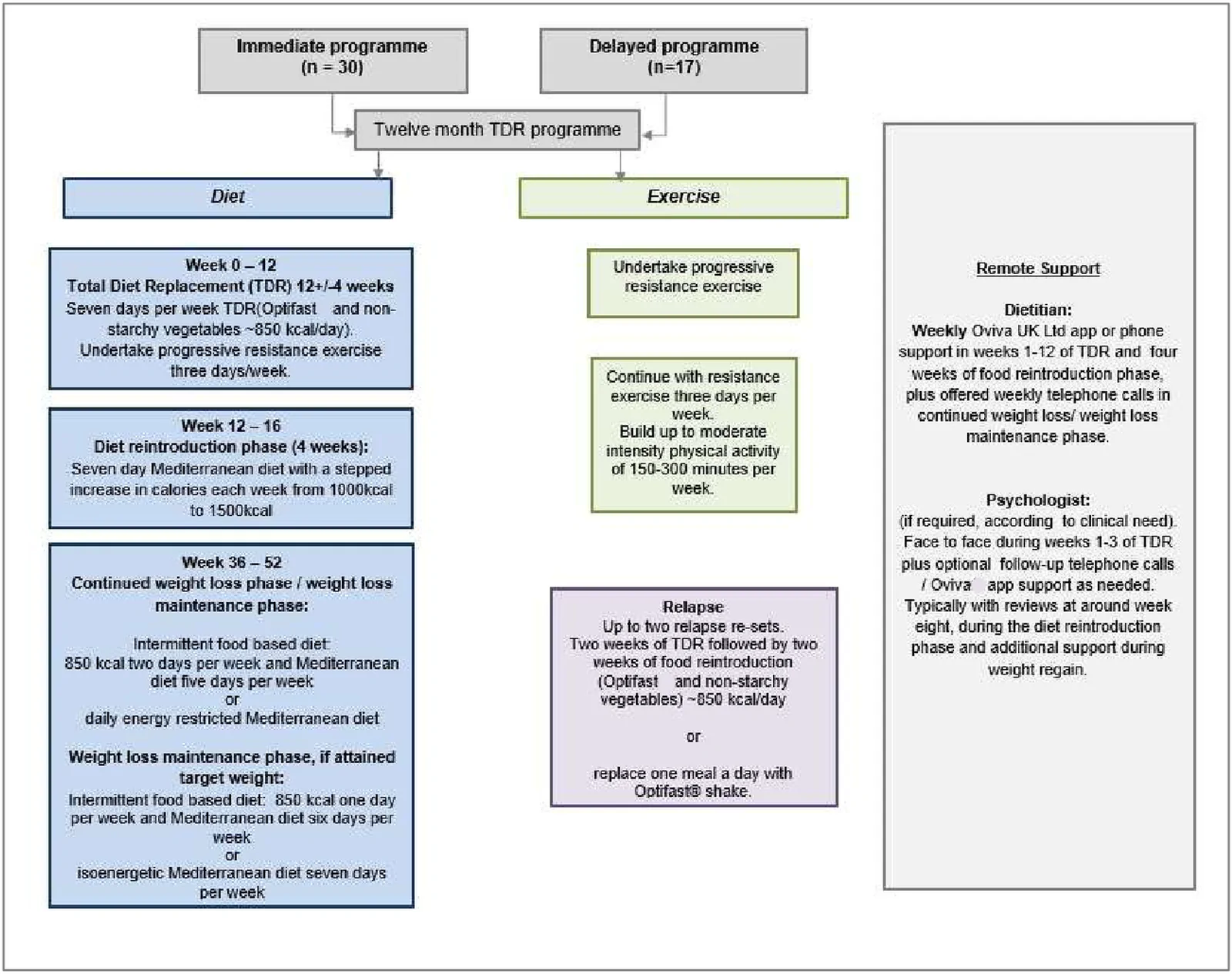
Schematic of 12-month TDR-based programme including dietary phases, relapse management, recommended exercise and remote support. TDR, total diet replacement.

In the first 12 weeks, participants followed a low-calorie diet replacement diet (850 kcal (3556 kJ)/day) which consisted of 3 sachets of Optifast and 5–8 portions of non-starchy vegetables per day. The subsequent 4 weeks involved a stepped food reintroduction; 1000 kcal (4184 kJ) in week 13, 1200 kcal (5021 kJ) in week 14, 1400 kcal (5858 kJ) in week 15 and 1500 kcal (6276 kJ) in week 16 which comprised Optifast and portion-controlled meals based on a Mediterranean diet.

From week 17 onwards, participants who had attained a weight loss of ≥15% and their target weight were asked to follow their choice of either a food-based isoenergetic intermittent diet (one 850 kcal (3556 kJ) day and 6 days of a Mediterranean diet per week) or food-based continuous weight maintenance diet (7 days of a portion controlled Mediterranean diet per week). The energy content of the diets was determined by the trial dietitian based on the Mifflin equation,^13^ multiplied by the metabolic equivalents for their self-reported activity levels.^14^ The Mediterranean diet provided 30% energy from fat (15% monounsaturated fatty acids, 8% polyunsaturated fatty acids, 7% saturated fatty acids), 25% energy from protein and 45% from low glycaemic load wholegrain carbohydrates. It included at least five portions of vegetables and two portions of fruit per day, low-fat dairy products, protein foods including fish, lean meat and pulses as described previously.^15^

Participants who had not achieved the trial weight loss goal of ≥15%, or who wished to lose more than 15%, were asked to follow a 25% energy-restricted weight loss diet that was either intermittent (two food based 850 kcal (3556 kJ) day and 5 days of a Mediterranean diet) or continuous (7 days of a Mediterranean diet).

### Physical activity

Physical activity was encouraged throughout all phases of the programme. The participant’s responses to the Physical Activity Readiness Questionnaire (Canadian Society for Exercise Physiology)^16^ assessed any contraindications to exercise. General practitioner clearance for physical activity was requested if required.

During the TDR phase, physically capable participants were asked to follow a progressive resistance exercise programme, comprising one to three sets of 8–15 repetitions of arm, leg and trunk resistance exercises, three times per week, over 12 weeks. Exercises were described in a written booklet and demonstrated through online videos (Physiotec, UK). Progression was tailored to their previous level of fitness and reviewed during their calls with the dietitian. Adherence to the exercise programme was from self-reporting to the trial dietitian and/or via the study app.

After the TDR phase, participants were encouraged to continue with the resistance exercises and build up to between 150 and 300 min of moderate intensity physical activity per week, that is, brisk walking to promote health and continued weight loss or weight loss maintenance.

### Behavioural dietetic and psychologist support

Participants received individualised advice and support from their allocated dietitian using motivational interviewing. The programme was delivered remotely using the Oviva app with either video or standard telephone call. The app facilitated written messages, self-monitoring of diet, weight, activity levels and an invitation to take part in peer-support messaging with other participants.

Support from the trial psychologist was offered to participants with high baseline scores for Patient Health Questionnaire-9 (PHQ-9)^17^ score of 10–14, or Generalised Anxiety Disorder (GAD-7)^18^ score of 10–14 and low self-efficacy evidenced by a Weight Efficacy Lifestyle Questionnaire Short-Form (WEL-SF) score of <45.^19^ Participants could be referred to the psychologist at any point throughout the study, should they meet these criteria, or experience difficulties at any time, including those entering the relapse programme. Psychological support was centred on motivational interviewing, cognitive–behavioural therapy, behavioural activation, mindfulness skills, distress tolerance skills, emotional regulation skills and acceptance and commitment theory considering values to make change meaningful and hooks and helpers’ that would help move towards committed action.

The psychological support involved three consultations at weeks three and eight of the TDR phase and during the food reintroduction period, with the aim of identifying psychological obstacles to adherence and problem solving throughout the diet. Psychological support was tailored to the needs of the individual and used a motivational interviewing style. Difficulties were formulated drawing on cognitive-behavioural and acceptance and commitment-based approaches, for example, considering values that would help to make change meaningful or ‘hooks and helpers’ that would help the individual move towards committed action. Specific issues disclosed to the psychologist were only shared with the rest of the multidisciplinary team with their agreement and if clinically relevant and where appropriate participants were signposted to NHS Talking Therapies.

### Relapse management for weight gain (weeks 12–52)

If participants regained ≥2 kg (from self-reported weight data), they were advised they could resume the 850 kcal (3556 kJ) TDR for 2 weeks followed by 2 weeks of diet reintroduction. They were also offered a booster call from the trial dietitian and additional support from the trial psychologist. TDR and dietitian/psychologist support were offered for the first two relapses and dietitian/psychologist support only for any subsequent relapses.

## Study outcomes

### Study uptake, retention and participant characteristics

Uptake and recruitment were recorded by the number of expressions of interest following mailshot, potentially eligible participants and the number of women randomised to the study following informed consent. Reasons for joining the study were recorded at the time of recruitment. Retention was defined through maintained contact with study staff, and whether participants completed the study, withdrew or became lost to follow-up. Descriptive statistics describe the characteristics of the study cohort.

### Changes in body weight and anthropometry

This was assessed in person at baseline and after 3 months, 6 months, 9 months and 12 months of the programme by research practitioners who were independent from programme delivery. Height was measured at baseline to the nearest millimetre, with the Frankfort horizontal plane, using a portable stadiometer (Chasmors, London). Body weight was measured to the nearest 0.1kg in light clothing, without shoes or socks, using calibrated bioimpedance scales (Tanita MC980MA Tanita Europe, Amsterdam, The Netherlands) using a standardised protocol.^20^ Waist circumference was measured across the umbilicus and hip circumference was measured over the participant’s underwear at the widest point over the buttocks to the nearest 0.5 cm.

### Adherence to TDR and exercise during weeks 1–12

Adherence to TDR and resistance exercise during weeks 0–12 of TDR was assessed from self-reports during their dietitian review phone call and/or via messages/recording on the Oviva app. Adherence to physical activity throughout the programme was assessed using the International Physical Activity Questionnaire (Short Form).^21^

### Fidelity of delivery of the programme

Fidelity of programme delivery was evaluated by the number of contacts with the dietitians, and the duration of dietitian time for each patient for both successful and unsuccessful contacts and the modality of contact, that is, telephone calls, video calls. We recorded the number of referrals to the study psychologist, and at which point during the study the participant was referred. Also, onwards referral to community mental health services at the end of study participation. Use of the Oviva app and its functions across the 12-month programme, that is, self-monitoring of diet, weight, activity levels and peer-support messaging, were recorded.

### Mental well-being, QoL, weight loss self-efficacy and binge eating

Participants were asked to complete questionnaires at baseline and at 3 months, 6 months, 9 months and 12 months to assess depression (PHQ-9), anxiety (GAD-7), depression, QoL (OWL-QoL),^22^ EQ-5D-health,^23^ self-efficacy for ability to lose weight (WEL-SF) and Binge Eating Scale (BES).^24^

### Adverse events

Adverse events potentially related to the TDR dietary programme were evaluated in both groups in week 1–12 of the study, when the immediate diet group were following TDR and the delayed group were following their normal diet. These included alopecia, constipation, biliary disorder (gallstone formation, colic, jaundice, cholecystitis), constipation, diarrhoea, hunger, gallstones, light-headedness, cold intolerance and dry skin,^25–27^ scored using Common Terminology Criteria for Adverse Events (V.5).^28^ Serious adverse events were recorded throughout the study.

### Statistics

The study was powered for the primary outcome, the change in cellular proliferation (Ki67) within the tissue biopsies, which will be reported elsewhere. This adherence paper reports baseline characteristics for the overall, immediate and delayed programme groups and uptake and retention to these groups. Since the immediate and delayed programme groups undertook the same 12-month TDR programme, study outcomes at 3, 6, 9 and 12 months of the programme are reported with descriptive statistics for the overall combined group. Data are presented as mean (SD) for normally distributed and median (IQR) for non-parametric continuous variables and number (percentage, %) for categorical variables. Data are presented, using all available data at each time point, with no imputation for missing data using Stata (V.18, Stata).

## Results

### Participant characteristics

The study population is described in table 1. The mean age was 40.5 (5.8) years and median (IQR) BMI 35.9 kg/m^2^ (33, 40.5). The randomised groups were of comparable age, weight and other demographics and were predominantly of White British ethnicity (87%). Most of the cohort were either living with a husband or partner (70.2%) and had at least one child under the age of 18 living at home (78.3%). The median Tyrer-Cuzick lifetime breast cancer risk score was 20.9% (IQR 16.8, 28.6), with the majority being at moderate risk of breast cancer as defined by NICE criteria (>17% lifetime risk).^29^ Most had at least GCSE/O-Level qualifications, with 40.4% of women being educated to degree level and 93.6% were in a form of paid employment. The cohort included participants from all quintiles of social deprivation, with most being from the least deprived quintiles 7–10 (61.7%).

**Table 1:** Baseline characteristics of study participants

	All participants joining the programme (n=47)	Immediate programme group (n=30)	Delayed programme group (n=17)
Age, years*	40.5 (5.8)	40.8 (5.5)	39.9 (6.5)
Ethnicity			
White	41 (87)	27 (90.0)	14 (82.4)
Black	3 (6)	2 (7)	3 (18)
Asian	2 (4)		
Mixed	1 (2)	1 (3)	
BMI (kg/m^2^)†	35.9 (33, 40.5)	36.2 (33.2, 39.1)	35.2 (32.4, 42.3)
BMI (>35 kg/m^2^)	26 (55.3)	17 (56.7)	9 (52.9)
Weight gain since age 18 years (kg)†	30 (22.1, 41.5)	30.6 (24.1, 36.8)	27.4 (21.4, 48.8)
Tyrer-Cuzick lifetime breast cancer risk (%)†	20.9 (16.8, 28.6)	22.8 (17.7, 29.1)	17.4 (16.6, 23)
Tyrer-Cuzick score >17%	34 (72.3)	23 (76.7)	11 (64.7)
Marital status			
Divorced	3 (6.4)	2 (6.7)	1 (5.9)
Household with others	5 (10.6)	4 (13.3)	1 (5.9)
Husband/partner	33 (70.2)	22 (73.3)	11 (64.7)
Single	6 (12.8)	2 (6.7)	4 (23.5)
Children 18 years or younger living at home			
0 1 2 3 4	36 (78.3) 10 (21.3) 6 (12.8) 25 (53.2) 4 (8.5) 2 (4.3)	23 (79.3) 5 (16.7) 6 (20) 16 (53.3) 2 (6.7) 1 (3.3)	13 (76.5) 5 (29.4) 0 (0) 9 (52.9) 2 (11.8) 1 (5.9)
Highest education			
No qualifications O Level/GCSE A-Level/Post-16 Degree Postgraduate	2 (4.3) 6 (12.8) 14 (29.8) 19 (40.4) 6 (12.8)	1 (3.3) 5 (16.7) 7 (23.3) 13 (43.3) 4 (13.3)	1 (5.9) 1 (5.9) 7 (41.2) 6 (35.3) 2 (11.8)
Employment status			
Part time Unemployed Voluntary work <30 hours Receiving welfare benefits	14 (29.8) 2 (4.3) 1 (2.1) 10 (21.7)	11 (36.7) 1 (3.3) 0 (0) 5 (17.2)	3 (17.6) 1 (5.9) 1 (5.9) 5 (29.4)
Deprivation quintile			
1–2 most deprived 3–4 5–6 7–8 9–10 least deprived	6 (12.8) 9 (19.1) 3 (6.4) 17 (36.2) 12 (25.5)	3 (10) 5 (16.7) 3 (10) 12 (40) 7 (23.3)	3 (17.6) 4 (23.5) 0 (0) 5 (29.4) 5 (29.4)

* Mean (SD)

† Median (Q1, Q3) and categorical as n (%) Indices of Multiple Deprivation 2015.

BMI, body mass index.

### Uptake and retention

Uptake and retention of study participants are shown in figure 2. Of the 897 patients registered with the Manchester University NHS Foundation Trust family history risk and prevention clinic contacted by mailshot, 92 expressed an interest in the study. An additional four expressed an interest following an internal digital communication to staff at the NHS Trust. At initial screening, 30 women were excluded as they did not meet the eligibility criteria and 14 declined to participate. 52 were deemed provisionally eligible and subsequently 47 were randomised as five were identified to be ineligible (figure 2). Ineligibility reasons included high PHQ-9 score at baseline, new diagnosis of T2DM, use of medicines linked to weight gain and perimenopausal with irregular menstrual cycles. 37 (79%) completed the study. Completers and those who withdrew, respectively, had a mean (SD) age of 41.4 years (5.7) vs 38.1 (5.8), baseline BMI of 36.2 (4.9) kg/m^2^ vs 40.0 (7.9) kg/m^2^ and 76% vs 90% had children aged 18 or younger living at home. No significance testing was performed due to the small sample size (online supplemental table 2).

**Figure 2:**
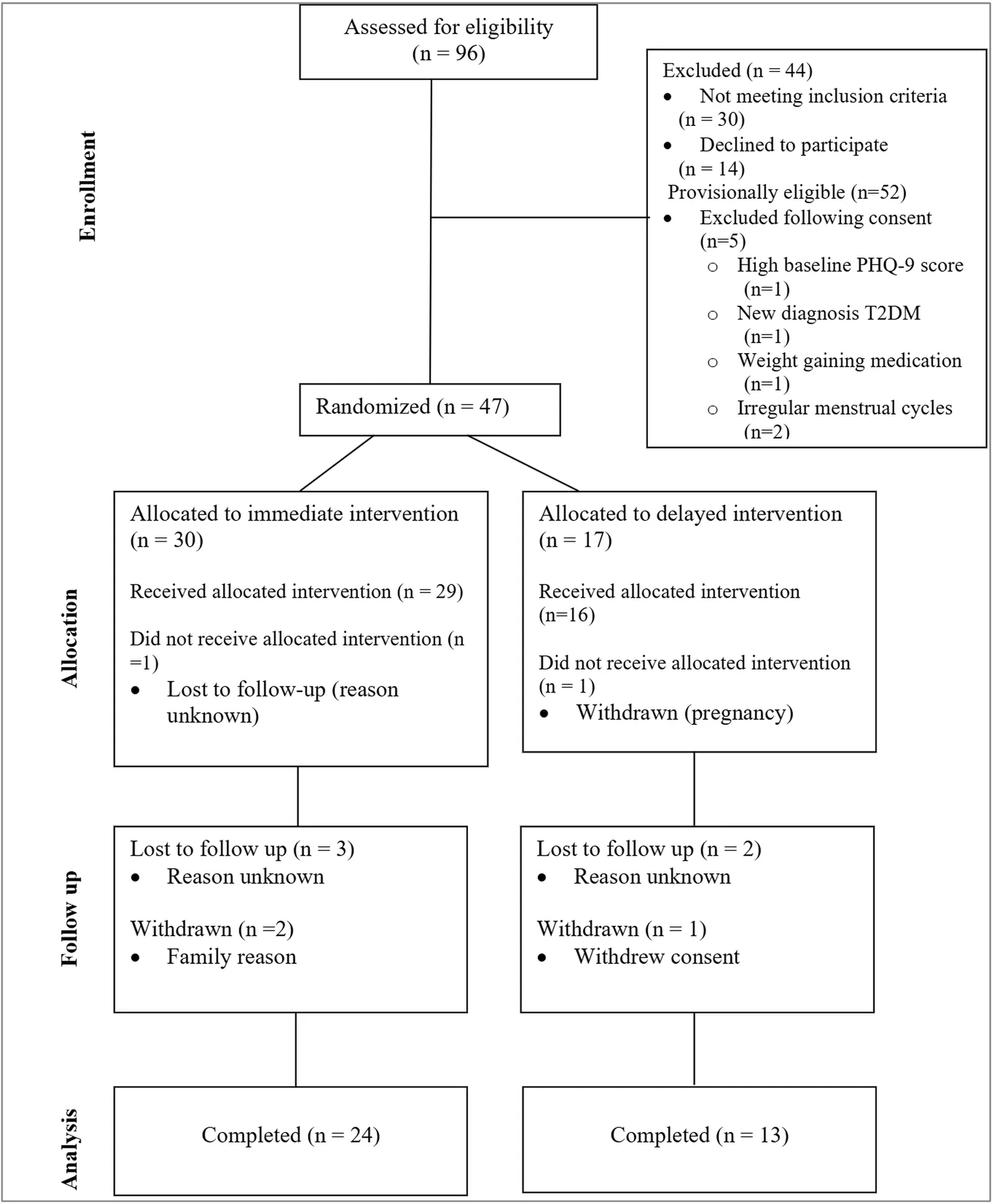
Consolidated Standards of Reporting Trials diagram for the PROBE-TDR study. PHQ-9, Patient Health Questionnaire-9; PROBE-TDR, Prevention Of Breast and Endometrial cancer using Total Diet Replacement; T2DM, type 2 diabetes mellitus.

### Participant reasons for joining the programme and motivation and confidence to achieve weight loss with the programme

The most common reasons for joining the research study were to achieve weight loss 32 (68%), increase energy and mobility 25 (53%) and boost self-confidence or happiness with appearance 13 (28%). Motivation to reduce their personal cancer risk was the least common reason stated 4 (9%), in spite of 73% having been informed they were above population level for breast cancer risk, and all were considered to be at increased risk of endometrial cancer related to their BMI (online supplemental table 3).

Thus, weight loss was an important motivator, but confidence that this could be achieved was lower; 16 (35%) were highly confident (scoring 5 out of 5) and 10 (22%) were moderately confident (3 out of 5) (online supplemental table 4).

### Weight loss and adherence to diet and physical activity

Weight loss in the immediate and delayed TDR groups are comparable and reported in figure 3. Changes in body weight, body composition and anthropometrics for all participants are reported in table 2. 45 (97%) completed the 3 months of TDR, 40 (85%) completed 6 months and 37 (79%) completed the 12-month programme. There was a median (IQR) reduction in weight of −10.8 kg (−13.8, −7.2) at 3 months and −11.0 (−16.4, −7.8) (figure 3). At 12 months, there were overall median reductions in body fat and fat-free mass, −3.5% (−5, −0.7) and −2.4 kg (−4.2, −0.8), respectively (table 2). The number of women in the cohort achieving >5.0% wt loss at 3 months, 6 months and 12 months, respectively, were 40 (88%), 31 (78%), 26 (70%) and those achieving ≥10.0% wt loss were 28 (62%), 22 (54%) and 17 (46%), respectively.

**Figure 3:**
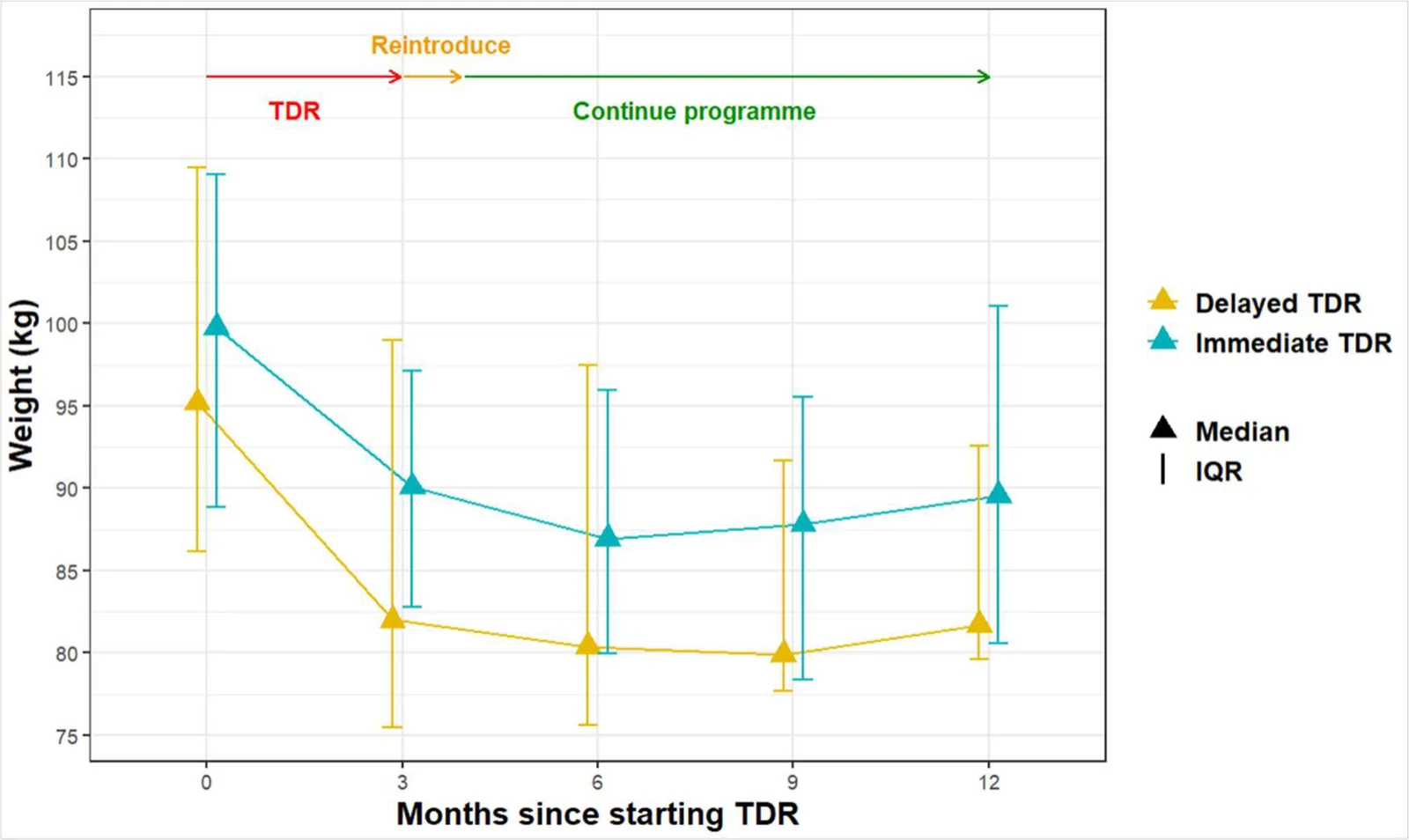
Weight change of participants in both immediate and delayed intervention groups across the 12-month TDR programme. Numbers of participants per group at each time point are displayed (blue=immediate intervention group, yellow=delayed intervention group). TDR, total diet replacement.

**Table 2:** Changes (per cent from baseline) in weight and body composition for all participants across the 12-month programme

	Baseline (n=47)	Change 3 months (n=45)	Change 6 months (n=41)	Change 9 months (n=35)	Change 12 months (n=37)
Weight (kg)	98.3 (88.2, 109.1)	−10.8 (−13.8, −7.2)	−11.5 (−15, −7.6)	−11.0 (−16.4, −7.8)	−8.4 (−13.9, −3.7)
Body fat (%)	43.7 (40.8, 46.1)	−3.0 (−4.3, −1.9)	−4.0 (−5.6, −1.8)	−4.5 (−5.5, −0.9)	−3.5 (−5, −0.7)
Fat-free mass (kg)	55.9 (51.4, 59.6)	−3.5 (−4.3, −2)	−2.6 (−3.7, −1.2)	−2.6 (−4.4, −1.6)	−2.4 (−4.2, −0.8)
Hip (cm)	119.5 (112.2, 129.2)	−7.5 (−10, −4.5)	−8.5 (−11, −4.5)	−7 (−11.4, −5)	−7.5 (−11.5, −4.5)
Waist (cm)	109.8 (102.1, 121.9)	−10.5 (−15.5, −6.5)	−11.5 (−17, −9)	−12.5 (−17.8, −9)	−13.0 (−17.5, −7)
		3 months	6 months	9 months	12 months
5.0%–9.9% wt loss	Number (%)	15 (33)	10 (24)	5 (14)	9 (24)
≥10.0% wt loss		28 (62)	22 (54)	23 (67)	17 (46)

Median (IQR).

Self-reported adherence to the prescribed low-calorie days during the TDR phase was 93% (IQR: 87%, 100%) (table 3). Reasons given for days off TDR included religious festivities, birthdays and celebrations, illness, stress, live events, premenstrual symptoms and holidays. Over the course of the programme, 19 (40.4%) participants required a reset because of weight regain, with the largest proportion, 46.9%, occurring between months 6 and 9. There was poor adherence to the resistance exercise programme. This was only achieved in 6 (1, 10) of the 13 weeks of TDR and median number of the prescribed sessions undertaken per week was 0 (IQR: 0, 6).

**Table 3:** Self-reported adherence to the TDR programme for all participants

Percentage of low-calorie days undertaken across the 12 weeks on TDR	93% (87, 100)
Key reasons for days off TDR, n (%)	Religious festivities 9 (26) Birthday/family celebration 9 (26) Unwell/COVID 6 (17) Holiday 6 (17) Stress/anxiety 4 (11) Bereavement 1 (3)
Diet resets as a result of weight regain	
Number of participants with TDR re-sets, n (%) of those starting the programme	19 (40.4)
Reset in months 3–6, n (%) still active in the programme	9 (21.0)
Reset in months 6–9, n (%) still active in the programme	15 (46.9)
Reset in months 9–12, n (%) still active in the programme	8 (21.1)
Adherence to resistance exercise during the initial 12-week TDR phase	
Number of weeks with resistance exercise during the 12-week TDR phase (IQR)*	6 (1,10)
Number of resistance exercise sessions per week (IQR)	0 (0,6)

Median (IQR).

* Resistance exercise programme comprised 1–3 sets of 8–15 repetitions of arm, leg and trunk exercises, three times per week during the initial 12-week TDR phase of the programme.

TDR, total diet replacement.

### Fidelity of delivery of the programme

Across the 12-month programme, there was a median 28 (25,33) attempted dietitian contact per participant, of which 89% were successful contacts (online supplemental table 5). A total of 13 hours and 24 min was used on 154 unsuccessful contacts across all participants. Contacts were made predominantly by video call, 276 (70%), but also by telephone, 117 (30%). A high proportion of the cohort were eligible to be referred to the trial psychologist (68%), with the majority identified at baseline (40%). The median (IQR) number of psychologist appointments per participant was 3 (1, 6). Of the women referred to the psychologist, 41% were previously known to mental health services and 22% were triaged to other mental health services during the programme. The majority of participants started using the Oviva app (n=43; 91%). Use declined over time, that is, median (IQR weight recording was 7 (0, 12) in the TDR phase and reduced to 0 (0, 2) between 9 and 12 months. Only 10 (21%) used the peer support chat function on the Oviva App. Oviva app use declined after the TDR phase (online supplemental table 5).

### QoL, mental health and self-reported physical activity

These measures are reported in table 4. There were numerical increases in median QoL (OWL-QOL and EQ5D) scores and a numerical reduction in median BES but no changes in the mental health or physical activity scales.

**Table 4:** Quality of life, mental health and physical activity measures across the 12-month programme

	Baseline	3 months	Change at 3 months	12 months	Change at 12 months
GAD-7	3 (1.2,6)	3 (1, 6)	−1 (−2, 1)	3.0 (1, 6.0)	−0.0 (−2.2, 2)
PHQ-9	4 (3,5.8)	3 (1.5, 6)	−1 (−3, 1)	3.5 (1, 6.8)	−1 (−2, 2.8)
WEL-SF	53.5 (40.5, 61)	57 (46, 66)	6 (−5, 14)	56.5 (49, 68)	4 (−3, 18.2)
EQ5D-Health	6.9 (5.5, 8)	7.5 (7, 8.2)	0.7 (0, 2)	7.5 (6, 8.5)	1 (0, 2)
OWL-QOL	47 (27.5, 61)	31.5 (13.2, 41)	−15 (−28.5, −2)	25 (11.8, 34.5)	−17 (−24.8, −7.8)
BES	12.5 (9, 17)	8 (6, 13)	−3 (−8, 1)	7.5 (4, 14)	−4 (−6.5, −0.8)
IPAQ/met hours per week	1144 (396, 1929)	1008 (589.9, 2982)	291 (−936, 1505.1)	1035 (462, 2784)	16 (−444, 521.5)

Median (IQR).

BES, Binge Eating Scale; GAD-7, Generalised Anxiety Disorder-7; IPAQ, International Physical Activity Questionnaire; PHQ-9, Patient Health Questionnaire-9; QOL, quality of life; WEL-SF, Weight Efficacy Lifestyle Questionnaire Short-Form.

### Adverse events

Adverse events were recorded in the initial 3 months of the study during TDR for the immediate programme group and during 3 months of normal diet for the delayed programme group to act as controls (online supplemental table 6). A total of 83 grade 1, 2 grade 2 and 1 grade 3 events were reported in the immediate programme group. The overall number of events was lower in the control group, with 31 grade 1, 3 grade 2 and no grade 3 events reported. The most common adverse events in both groups were constipation reported in the immediate TDR versus the delayed TDR control group (55.2% vs 18.8%), diarrhoea (34.5% vs 25%) and headache (65.5% vs 31.2%).

## Discussion

The study suggests TDR could be a viable weight loss programme for women at increased risk of breast, endometrial and other obesity-related cancers, since many of the cohort achieved weight loss of >5% and 10%, which have been associated with cancer risk reduction.^1^ Rates of retention and weight loss (>5% and >10%) at 12 months are comparable with those reported in previous 12-month TDR programmes among individuals with general obesity^30 31^ and those with obesity and type 2 diabetes.^32^ Notably, adherence was not higher in this population who knew they were at increased cancer risk. This aligns with the observation that cancer risk was not a key motivator for joining the trial, which was mainly driven by a desire to lose weight, be fitter and feel happier with their appearance. Previous work among high-risk women has highlighted a low belief that weight loss can really impact on cancer risk.^33^

The higher numbers of dropouts from the intervention among younger women, including those with children living at home, align with lower adherence and weight loss outcomes among mothers with children in the literature.^34 35^ This is thought to be linked to competing pressures on their time, family dietary preferences and issues with incorporating healthy eating into the home environment. Our data suggests these issues may impact among people following a TDR diet programme, which requires them to have different meals to the rest of the family during the TDR phase. Future interventions should explore whether elements of family interventions could be integrated into the TDR programme to improve these outcomes for parents of children living at home.

Dietary adherence was good, however there was low adherence to the resistance exercise programme which participants were advised to follow during the TDR phase. Despite regular reinforcement from the dietitian during the TDR phase, self-reported physical activity did not change across the 12-month programme. The programme was delivered remotely on telephone/video calls. Remote delivery has proven efficacy for dietary change and weight loss,^36^ but there is less robust evidence that home-based programmes can increase physical activity, particularly resistance exercise.^37 38^ Higher adherence has been reported for supervised resistance exercise during TDR.^39^

It is important to note that this high cancer risk cohort with obesity had a relatively high psychological morbidity, and many received psychological support within the programme. Psychological support is recognised as an important component of weight management and with low-calorie diets in other clinical settings.^40^ The prevalence of eating disorders among those with a family history of cancer is not known. Rates are likely to be as high or higher than other cohorts since some of the population will have experienced early life bereavement, which has been associated with the development of bulimia nervosa and binge eating disorder.^41^

Strengths of the study are a novel use of TDR in a population at high risk of cancer, with 12-month data. Limitations include the cohort being a self-selected group who had joined a breast and endometrial mechanistic biopsy study. The cohort had limited ethnic diversity, including mainly white women and only 13% from different ethnic groups. However, the cohort recruited had a range of social deprivation scores, with 20% receiving welfare benefits and 60% scoring in the lowest 40% of deprivation scores.

The low recruitment rate of 10% aligns with previous recruitment rates to other weight loss intervention trials in our high-risk clinic, as well as recruitment rates to TDR in a general population.^30 42^ We used an untargeted mailshot to provisionally eligible women, many of whom would not have met the eligibility criteria for BMI, which was not available on the hospital database. The mailshot will also have skewed the population to those most highly motivated, however the clinic has an active research presence, and many clinic attendees are happy to be approached, compared with the general hospital population. The study was launched in the COVID-19 pandemic. COVID restrictions did not prevent participants from attending study appointments which benefitted from the remote components of the study design. This may, however, have prevented participants from joining due to the risk perception of COVID-19 in the UK population at that time.

A further limitation is the high input required for multidisciplinary support alongside the dietary programme, that is, the dietitian contact time and the high referral rate to the study psychologist, which would have a significant impact on scalability of the intervention.

This support time is likely to exceed what was actually required since contact time was protocol driven was not necessarily responding to the needs of the participants. Future work could involve a study using stepped support TDR interventions which respond to participants’ needs, which have been shown to be less resource intensive with other weight loss interventions.^43^ Another limitation of the study is the lack of long-term follow-up. Weight regain is common with many dietary interventions,^44^ therefore we are unable to comment on weight loss durability from this analysis.

This study adds to the body of data to support remotely delivered low calorie diet interventions. There was modest use of the self-recorded functions on the app, but low engagement with the peer support chat function which is consistent with other studies.^45^ Peer support is potentially important for sustained weight loss and future work should explore ways to facilitate this.

Future work could explore how best to maintain weight loss beyond the 12-month programme which may be required for maintained cancer risk reduction. Recent data 2 years post a 12-month TDR programme reported maintained weight loss of >5% (33%) and 10% (19%).^46^ The recently approved GLP-1 agonist therapies are also used (in conjunction with lifestyle changes) to achieve weight loss of greater than or equal to 10%. However, their use is licensed for individuals with BMI of ≥35 with a weight-related comorbidity, that is, hypertension or hyperlipidaemia.^47^ Only six (13%) of the cohort herein would meet these eligibility criteria.

TDR programmes are currently used in the UK NHS for remission of type 2 diabetes and can be used in tier three weight management services for those with BMI >35 and comorbidities. Owing to the recent expansion of use of GLP-1 agonists, a detailed health economic evaluation of TDR against GLP-1 is now warranted. The success of the TDR programme among high-risk women in this study indicates that low calorie diet programmes could expand beyond these current services to include those identified at increased risk within high-risk cancer family history clinics. The low recruitment rate, absence of long-term control and poor exercise adherence limit the generalisability of the findings. Controlled trials with extended follow-up are required before recommending routine implementation of TDR in high-risk clinics.

## Supplementary material

10.1136/bmjopen-2026-119498 online supplemental file 1

## Data Availability

All data relevant to the study are included in the article or uploaded as supplementary information.
