# Supplementary material for: Adherence and weight loss with a total diet replacement programme for increased cancer risk associated with obesity: a UK cohort study
Source: BMJ Open. 2026 Sep 28;16(9):e119498. doi: 10.1136/bmjopen-2026-119498 (PMC13629845; doi:10.1136/bmjopen-2026-119498)
Supplement: Supplementary file 1 [file bmjopen-2026-119498.reviewer_comments.pdf]

PEER REVIEW HISTORY

BMJ Open publishes all reviews undertaken for accepted manuscripts. Reviewers are asked to complete a checklist review form and are provided with free text boxes to elaborate on their assessment. These free text comments are reproduced below.

ARTICLE DETAILS

Title (Provisional)

Adherence and weight loss with a total diet replacement programme for increased cancer risk associated with obesity, a UK cohort study

Authors

Clarke, Helen; Lombardelli, Cheryl; Barrett, Emma; Wisely, Julie; Sellers, Katharine; Issa, Basil; Howell, Anthony; Davidson, Emma J; Howell, Sacha; Harvie, Michelle

---

VERSION 1 - REVIEW

Reviewer 1

Name Alexandria, Shaina J.

Affiliation Northwestern University Feinberg School of Medicine, Department of Preventive Medicine (Biostatistics)

Date 16-Apr-2026

COI None

This project evaluates the feasibility, preliminary efficacy, and acceptability of a total diet replacement (TDR) intervention in a sample of pre-menopausal obese women at increased risk for breast or endometrial cancer. This is a secondary data analysis of data from a trial and is intended to evaluate only the delivery and preliminary efficacy of the dietary intervention, which consisted of a 12-week weight loss phase, a 4-week transition phase, and a maintenance phase for a total of 12 months of follow-up. Since both study arms eventually received the intervention, this paper reports on findings from the pooled sample.

General comments:

While it seems that a primary aim of this paper was to describe changes in metrics over the 12-month study period and differences between the completers and non-completers, no statistical analyses were conducted to evaluate the differences. If this is the goal, appropriate statistical tests should be run and the p-value or confidence intervals should be reported. This is true of both the differences from baseline to end of

follow-up (e.g., Wilcoxon signed-rank test for difference in medians for paired data) and the differences between completers and non-completers (e.g., t-test for difference in means in independent samples). Otherwise, please omit any language that discusses group differences, since no evidence is provided that differences were due to more than chance. As is, the discussion of differences is discordant with the analysis conducted.

Specific comments:

The claim that this intervention is effective for weight loss is not justified. From abstract, "Median 36 (interquartile range) weight was significantly reduced at all time points; -10.8kg (37 13.8, -7.2) at 3-months, -11.5kg (-15, -7.6) at 6-months, -11.0kg (-16.4, -7.8) at 938 months and -8.4kg (-13.9, -3.7) at 12-months." From main paper: Line 354 "The study suggests TDR is a viable weight loss programme for women at increased risk of 355 breast, endometrial and other obesity related cancers, with significant numbers achieving 356 weight loss of > 5% and 10%, which have been associated with cancer risk reduction(1)." These differences must be quantified with measures of uncertainty. Additionally, since these estimates are not relative to a control group and do not account for loss-to-follow-up, reported differences from baseline to end of follow-up should be interpreted cautiously.

Since there is potential evidence of differential loss to follow-up by baseline BMI (Supplementary table 2) and around a quarter of the sample dropped out, consider sensitivity analyses using baseline observation carried forward to evaluate whether findings (i.e., differences in weight, waist circumference) are robust to the presumed MNAR mechanism if the primary goal is to estimate efficacy of the TDR protocol for weight loss. However, this may be beyond the scope of this paper.

It is mentioned that per-protocol analyses were conducted, but more clarity is needed on how this was operationalized. Are all the differences reported in completers only? Does the definition of "completers" depend on the survey/study time point?

The flow chart in Figure 2 indicates new diagnosis of T2DM is an exclusion criterion; however this is not mentioned in the study design section, and Supplemental Table 1 indicates that participants with T2DM can be in the study. Please clarify.

Figure 3 caption does not indicate what the points and error bars represent.

Reason for joining the study is not described in the Study Outcomes section of the Methods, but is reported as an outcome in the Results section.

**Reviewer**	**2**
**Name**	**Hoegl, Jorge**

**Affiliation**	**Centro Clinico Profesional, Gyne Onc**
**Date**	**17-May-2026**
**COI**	**None**

The manuscript addresses a relevant and novel topic: the feasibility of a 12-month total diet replacement (TDR) program in a population at high oncological risk. However, I would like to make several comments, particularly regarding the Methods section, which could contribute to an improved outcome for the manuscript.

The title is adequate but long; it could be simplified. The conclusion ("TDR is a viable program") is too robust given the absence of a 12-month control group. I suggest modifying it to be more consistent with the reality of the manuscript. Furthermore, the "Strengths and limitations" section should explicitly include the low recruitment rate (only 92 of 897 contacted, 10%).

Introduction: I suggest that the authors add a more explicit and justifiable statement of the knowledge gap: there are no data on adherence, retention, and psychosocial outcomes of TDR in this specific population.

Methods:

This is not an RCT for the 12-month outcomes. After the initial period, the data are presented as a case series without a control. The authors should rename the study as an "analysis of an uncontrolled prospective cohort" for this manuscript and justify this. Add a sentence indicating that this study is a secondary analysis (statistical power was for the primary outcome of tissue biomarkers). It should also be clarified that the control group was only valid for the first 3 months, and that from then onward, both groups received TDR (de facto single-arm design).

It is reported that the median number of resistance exercise sessions was 0 per week. There is no description of whether any strategies were implemented to improve this. An explanation of why this component of the intervention failed should be added.

Results:

- Table 2: The row for "≥10.0% weight loss" at 12 months (46%) is it miscalculated? According to the text, 17/37 = 46%, which is correct. However, the number of patients achieving ≥15% weight loss, which would be clinically relevant for risk remission, is missing. I suggest adding it.

- Line 265 (page 12): "completers appeared to have a higher mean age of 41.4 years (5.7) vs 38.1 (5.8)" – it is not indicated that this difference was statistically evaluated. A comment should be added that no significance testing was performed due to the small sample size.

- The high proportion of referrals to a psychologist (68%) and the mean contact time with dietitians (7.6 hours per participant) should be presented as a limitation to scalability, not merely as a descriptive finding.

Discussion:

- The low recruitment rate (10% of those contacted) is not discussed. This indicates massive selection bias: only highly motivated women participated. A paragraph should be added in the limitations section.

- The effect of the COVID-19 pandemic on adherence and measurements is not mentioned, although it is hinted at in the protocol. If there was no impact, this should be stated; if there was, it should be quantified.

- Lack of long-term follow-up beyond 12 months is a critical limitation, especially because the DROPLET study (reference 36) is cited, which showed weight regain at 3 years. The authors should comment that this study cannot evaluate durability.

- To what is the difficulty of remote programs attributed? This should be explained. At a minimum, it should be suggested that future studies include live supervision or incentives.

Conclusion: The low recruitment rate, absence of long-term control, and poor exercise adherence limit the generalizability of the findings. Controlled trials with extended follow-up are required before recommending routine implementation in high-risk clinics. This must be included; it is not consistent with the results to conclude that this program is a viable alternative.

VERSION 1 - AUTHOR RESPONSE

Reviewer 1	
While it seems that a primary aim of this paper was to describe changes in metrics over the 12-month study period and differences between the completers and non completers, no statistical analyses were conducted to evaluate the differences. If this is the goal, appropriate statistical tests should be run and the p-value or confidence intervals should be reported. This is true of both the differences from baseline to end of follow-up (e.g., Wilcoxon signed-rank test for difference in medians for paired data) and the differences between completers and non-completers (e.g., t-test for difference in means in independent samples). Otherwise, please omit any language	*This is a good point.*<br>*The protocol did include complete-case linear modelling for biomarker markers from baseline to 3 months to show the control group change at 3m and the intervention effect (i.e. the additional effect of intervention) (which will be published elsewhere).*<br><br>*Given the small sample size, we had not stated we would conduct statistical testing for the secondary 12-month programme endpoints reported herein. We have therefore amended the language used as suggested in the abstract and conclusion to remove references to significant changes and TDR being a viable programme for this population*<br>For example:<br><br>1. TDR could be a weight loss programme for women at increased risk of breast and endometrial cancers related to obesity.

that discusses group differences, since no evidence is provided that differences were due to more than chance. As is, the discussion of differences is discordant with the analysis conducted.	2. Likewise rephrased results for completers/ non completers in the abstract and results (line 288 in results)<br><br>Revised sentence (line 36): Completers and those who withdrew respectively had a mean (sd) age of 41.4 years (5.7) vs 38.1 (5.8) baseline BMI of 36.2 (4.9) kg/m^2 vs 40.0(7.9) kg/m^2 and 76% vs 90% had children 18 or younger living at home
The claim that this intervention is effective for weight loss is not justified. From abstract, "Median 36 (interquartile range) weight was significantly reduced at all time points; 10.8kg (37 13.8, -7.2) at 3-months, -11.5kg (-15, -7.6) at 6-months, -11.0kg (-16.4, -7.8) at 938 months and -8.4kg (-13.9, -3.7) at 12-months." From main paper: Line 354 "The study suggests TDR is a viable weight loss programme for women at increased risk of 355 breast, endometrial and other obesity related cancers, with significant numbers achieving 356 weight loss of > 5% and 10%, which have been associated with cancer risk reduction(1)." These differences must be quantified with measures of uncertainty. Additionally, since these estimates are not relative to a control group and do not account for loss-to-follow-up, reported differences from baseline to end of follow-up should be interpreted cautiously.	*This statement has been revised as requested in the absence of statistical evidence*<br><br>The study suggests TDR is a potential weight loss programme for women at increased risk of breast, endometrial and other obesity related cancers, Since many of the cohort achieved weight loss of > 5% and 10%, which have been associated with cancer risk reduction.
Since there is potential evidence of differential loss to follow-up by baseline BMI (Supplementary table 2) and around a quarter of the sample dropped out, consider sensitivity analyses using baseline observation carried forward to evaluate whether findings (i.e., differences in weight, waist circumference) are robust to the presumed MNAR mechanism if the primary goal is to estimate efficacy of the TDR protocol for weight loss. However, this may be beyond the scope of this paper.	As pre the previous comment we have not conducted further statistics due to the small sample size.

It is mentioned that per-protocol analyses were conducted, but more clarity is needed on how this was operationalized. Are all the differences reported in completers only? Does the definition of "completers" depend on the survey/study time point?	This is clarified in the methods section line 260.<br>Data are presented per protocol, using all available data at each time point, with no imputation for missing data
The flow chart in Figure 2 indicates new diagnosis of T2DM is an exclusion criterion; however this is not mentioned in the study design section, and Supplemental Table 1 indicates that participants with T2DM can be in the study. Please clarify.	Revised in methods, Line 112.<br>Patients with existing type 2 diabetes mellitus (T2DM) were eligible for inclusion, however we excluded those found to have a potential new diagnosis of T2DM on screening bloods.
Figure 3 caption does not indicate what the points and error bars represent.	Figure amended and legend updated.<br>Data points represent median with interquartile range.
Reason for joining the study is not described in the Study Outcomes section of the Methods, but is reported as an outcome in the Results section.	Revised in methods, line 208.<br>Reasons for joining the study were recorded at time of recruitment.
Reviewer 2	
The title is adequate but long; it could be simplified.	Revised, see earlier comment.
The conclusion ("TDR is a viable program") is too robust given the absence of a 12-month control group. I suggest modifying it to be more consistent with the reality of the manuscript.	Reworded this phrase throughout the manuscript.<br>TDR could be a weight loss programme for women at increased risk of breast and endometrial cancers related to obesity.
Furthermore, the "Strengths and limitations" section should explicitly include the low recruitment rate (only 92 of 897 contacted, 10%).	Revised as per earlier comment, line 64.<br>Added to the discussion line 430.
Introduction: I suggest that the authors add a more explicit and justifiable statement of the knowledge gap: there are no data on adherence, retention, and psychosocial outcomes of TDR in this specific population.	Revised, line 80.<br>However, within this specific population there is no data on adherence, retention, psychosocial outcomes or efficacy.
This is not an RCT for the 12-month outcomes. After the initial period, the data are presented as a case series without a control. The authors should rename the study as an "analysis of an uncontrolled	Revised throughout manuscript.

prospective cohort" for this manuscript and justify this.	
Add a sentence indicating that this study is a secondary analysis (statistical power was for the primary outcome of tissue biomarkers). It should also be clarified that the control group was only valid for the first 3 months, and that from then onward, both groups received TDR (de facto single-arm design).	Revised. Line 98<br>This cohort study reports secondary outcomes (adherence, changes in wellbeing and eating behaviours scores, in addition to fidelity of delivery of the programme) as the RCT was powered for reduction in tissue biomarkers. The control group is valid for the first 3-month analysis then both groups receive the full TDR intervention programme.
It is reported that the median number of resistance exercise sessions was 0 per week. There is no description of whether any strategies were implemented to improve this. An explanation of why this component of the intervention failed should be added.	We had already stated in the discussion. The low adherence to exercise is likely as the programme was remotely delivered & not supervised<br>This has been expanded in the discussion, Line 409. Despite regular reinforcement from the dietitian during the TDR phase.
Table 2: The row for "≥10.0% weight loss" at 12 months (46%) is it miscalculated? According to the text, 17/37 = 46%, which is correct. However, the number of patients achieving ≥15% weight loss, which would be clinically relevant for risk remission, is missing. I suggest adding it.	Thank you for the suggestion to add weight loss of > 15%. We have opted to just keep the 5 &10% & not include the 15%.<br>To the authors knowledge. There is no evidence that > 15% weight loss is specifically related to cancer risk reduction.<br>Although we acknowledge a recent retrospective study showed a linear relationship per 1% weight loss implying 15% would be associated with a greater reduction than 5 or 10% but 15% is not a specific cut off for risk reduction<br>https://pmc.ncbi.nlm.nih.gov/articles/PMC13032047/
Line 265 (page 12): "completers appeared to have a higher mean age of 41.4 years (5.7) vs 38.1 (5.8)" – it is not indicated that this difference was statistically evaluated. A comment should be added that no significance testing was performed due to the small sample size.	Revised.<br>Line 293. No significance testing was performed due to the small sample size
The high proportion of referrals to a psychologist (68%) and the mean contact time with dietitians (7.6 hours per participant) should be presented as a limitation to scalability, not merely as a descriptive finding.	Added as a limitation to the study in the discussion Line 432-440.<br>A further limitation is the high input required for multi-disciplinary support alongside the dietary programme i.e. the dietitian contact time and the high referral rate to the study psychologist which would have a significant impact upon scalability of the intervention This support time is likely to exceed what was actually required since contact time was protocol driven and was not necessarily responding to the needs of the participants. Future work could study stepped support TDR interventions which respond to patient

	needs, which have been shown to be less resource intensive with other weight loss interventions.
The low recruitment rate (10% of those contacted) is not discussed. This indicates massive selection bias: only highly motivated women participated. A paragraph should be added in the limitations section.	Added to discussion<br>Line 430<br>The low recruitment rate of 10% aligns with previous recruitment rates to other weight loss intervention trials in our high-risk clinic. Aswell as recruitment rates to TDR in a general population.
The effect of the COVID-19 pandemic on adherence and measurements is not mentioned, although it is hinted at in the protocol. If there was no impact, this should be stated; if there was, it should be quantified.	Discussion added, line 436<br>The study was launched within the COVID-19 pandemic and restrictions did not prevent participants from attending study appointments and benefitted from the remote components of the study design. This may, however, had prevented participants from joining due the risk perception of COVID-19 in the UK population at that time.
Lack of long-term follow-up beyond 12 months is a critical limitation, especially because the DROPLET study (reference 36) is cited, which showed weight regain at 3 years. The authors should comment that this study cannot evaluate durability.	added in limitations.<br>Added to the discussion Line 449.<br>Another limitation of the study is the lack of long-term follow-up. Weight regain is common with dietary interventions (44), therefore we are unable to comment upon weight loss durability from this analysis.
To what is the difficulty of remote programs attributed? This should be explained. At a minimum, it should be suggested that future studies include live supervision or incentives.	Remote programmes are effective for dietary adherence but much less so for promoting physical activity and resistance exercise. This was covered in the discussion.<br>The programme was delivered remotely on telephone/video calls. Remote delivery has proven efficacy for dietary change and weight loss (37), but there is less robust evidence that home based programmes can increase physical activity particularly resistance exercise (38,39).Higher adherence has been reported for supervised resistance exercise during TDR (40).
The low recruitment rate, absence of long-term control, and poor exercise adherence limit the generalizability of the findings. Controlled trials with extended follow-up are required before recommending routine implementation in high-risk clinics. This must be included; it is not consistent with the results to conclude that this program is a viable alternative.	We have added this statement at the end of the discussion:<br>The low recruitment rate, absence of long-term control, and poor exercise adherence limit the generalizability of the findings. Controlled trials with extended follow-up are required before recommending routine implementation of TDR in high-risk clinics.

VERSION 2 - REVIEW

Reviewer 1

Name Alexandria, Shaina J.

Affiliation Northwestern University Feinberg School of Medicine, Department of Preventive Medicine (Biostatistics)

Date 22-Jul-2026

COI

I thank the authors for their responsiveness to the comments provided previously. The reporting of statistics now aligns with the descriptive nature of the manuscript. I have two remaining suggestions to improve the paper:

1. In lines 250 and 300, the authors mention that results are presented "per-protocol." Typically, per-protocol analyses stratify based on whether the assigned protocol was followed. My impression of the meaning in this context is that the results presented represent data from the TDR phase for each treatment group. If this is an accurate description, please rewrite eliminating the term "per-protocol." If the results are actually reported by protocol compliance, please clarify that and add rational for why this was chosen.

2. Line 350: "There were improvements in quality of life (OWL-QOL and EQ5D and reductions in the binge eating scale but no changes in the mental health or physical activity scales." Since these changes were not statistically quantified, please remove language "improvements" and "reductions."

VERSION 2 - AUTHOR RESPONSE

Reviewer 1	
1. In lines 250 and 300, the authors mention that results are presented "per-protocol." Typically, per-protocol analyses stratify based on whether the assigned protocol was followed. My impression of the meaning in this context is that the results presented represent data from the TDR phase for each treatment group. If this is an accurate description, please rewrite eliminating the term "per-protocol." If the results are actually reported by protocol compliance, please clarify that and add rational for why this was chosen.	Revised.<br><br>Data are presented ~~per protocol~~ using all available data at each time point, with no imputation for missing data using Stata (version 18, StataCorp LLC, Texas, USA).<br><br>~~Per protocol~~ Changes in body weight, body composition and anthropometrics for all participants are reported in Table 2.
2. Line 350: "There were improvements in quality of life (OWL-QOL and EQ5D and reductions in the binge	Revised.

eating scale but no changes in the mental health or physical activity scales." Since these changes were not statistically quantified, please remove language "improvements" and "reductions."	There were numerical increases in median quality of life (OWL-QOL and EQ5D) scores and a numerical reduction in median binge eating scale but no changes in the mental health or physical activity scales.
