# Supplementary material for: Adherence and weight loss with a total diet replacement programme for increased cancer risk associated with obesity: a UK cohort study
Source: BMJ Open. 2026 Sep 28;16(9):e119498. doi: 10.1136/bmjopen-2026-119498 (PMC13629845; doi:10.1136/bmjopen-2026-119498)
Supplement: Supplementary file 2 [file bmjopen-16-9-s001.docx]

Supplementary information

| Supplementary Table 1: Inclusion and exclusion criteria of PROBE-TDR study | |
| --- | --- |
| Inclusion | Exclusion |
| Women aged 30–50 years. | Personal history of BC or EC or pre-invasive breast disease. |
| BMI ≥30 kg/m^2^ or ≥27.5 kg/m^2^ in high risk ethnic populations | Hormonal contraceptive use in the preceding 3 months. |
| Premenopausal, with regular menstrual cycles. | Preventative tamoxifen or anti-progestin therapy within the last 6 months. |
| Ability to use the Oviva UK app OR access to a telephone. | Known carrier of the BRCA1 or BRCA2 gene. |
| Willing to follow the TDR programme using Optifast products. | Confirmed pregnant at screening, planning pregnancy in the next 12 months or current breast feeding. |
| Maintain non-hormonal contraception (barrier or abstinence) until all biopsies completed. | Taking prohibited medications including warfarin or novel anticoagulants, low molecular weight heparin or equivalent anticoagulants, antipsychotic medication, antidiabetic medication other than metformin, orlistat or other pharmacological treatments for weight loss and steroids (more than 20 mg daily of prednisolone or its equivalent). |
| Type 2 diabetes mellitus on diet control±metformin can be included. | Previous bariatric surgery for weight loss including gastric bypass and sleeve gastrectomy. |
| Participants must be able to read, understand and communicate in English. | Known hypersensitivity to any of the Optifast® ingredients (eg, fish, milk, soy) or lactose intolerance. |
|  | Substance abuse or harmful alcohol use as indicated by a score of 16 or above on the Alcohol Use Disorders Identification Test (AUDIT). |
|  | Diagnosis of an eating disorder, or patients with severe binge eating assessed by a score of 27 or more on the Binge Eating Scale. |
|  | Severe depression assessed by a score of 15 or more on the Patient Health Questionnaire-9 (PHQ-9). |
|  | Severe anxiety assessed by a score of 15 or more on the General Anxiety Disorder (GAD-7) questionnaire. |
|  | Participants with psychiatric or physical comorbidity or scheduled for major surgery, which in the opinion of the treating medical physician, or the chief investigator (CI), would compromise their safety or adherence to the study. |

| Supplementary Table 2: Characteristics of participants completing the programme and those withdrawing from the programme | | |
| --- | --- | --- |
|  | Completer  n = 37 | Withdrawn / loss to follow up  n=10 |
| Age – years * | 41.1 (5.7) | 38.1 (5.8) |
| Baseline BMI* | 36.1 (4.9) | 40.0(7.9) |
| Estimated lifetime breast *cancer risk – Tyrer Cuzick | 22.5 (8.2) | 21.3 (8.2) |
| Deprivation quintile**  1-2 most deprived  3-4  5-6  7-8  9-10 least deprived | 24.0  40.5  5.4  21.6  8.0 | 30.0  20.0  10.0  10.0  30.0 |
| Ethnicity**  White  Black/ Asian / Mixed | 84  16 | 100  0 |
| Has children < 18 years **  living at home | 76% | 90% |

*Mean (sd) ** N (%)

| Supplementary Table 3: Self-reported motivations for joining the total diet replacement research programme | |
| --- | --- |
|  | Number citing response |
| To achieve their weight loss target | 32 (68) |
| To have more energy / mobility | 25 (53) |
| To improve their appearance/ self-confidence / clothes fitting better | 13 (28) |
| To change their eating behaviours/better control over diet | 10 (21) |
| To contribute to cancer research | 5 (11) |
| To reduce personal breast cancer risk | 4 (9) |

N(%).

| Supplementary Table 4: Self- reported confidence and motivation to achieve weight loss at the start of the TDR programme (n = 46) | | | | | |
| --- | --- | --- | --- | --- | --- |
| Confidence/Motivation Score | 1  (Low) | 2 | 3 (Moderate) | 4 | 5  (High) |
| How important is achieving your weight loss aim to you ? | 0(0) | 2 (4) | 4 (9) | 14 (30) | 26 (57) |
| How confident you can achieve your weight loss aims during the programme? | 0(0) | 0(0) | 10 (22) | 20 (43) | 16 (35) |
| How ready are you to make the necessary changes? | 0(0) | 0(0) | 3 (7) | 19 (41) | 24 (52) |

N(%).

| Supplementary Table 5: Dietitian and Psychologist support during the programme for all participants (n=46) | |
| --- | --- |
| Dietitian support | |
| Total number of attempted contacts across the 12-month programme | 28 (25,33) |
| Total dietitian time per participant (hours) | 7.6 (6.6,10.6) |
| Total time for successful contacts (hours) | 7.5 (6.2, =9.9) |
| Total time for unsuccessful contacts (hours) | 0.1 (0.0,0.6) |
| Types of contacts across the programme |  |
| Message on the Oviva app | 21 (16,27) |
| Video call | 4.5 (3.0,8.0) |
| Phone call | 1 (1,4) |
| Email | 0 (0,1) |
| Face to face | 0 (0,1) |
| Psychologist support | |
| Number of trial participants referred to trial psychologist | 32 (68) |
| Number of appointments (average per person referred) | 3 (1,6) |
| Timing of psychologist referral in the programme |  |
| Baseline | 19 (41) |
| During total diet replacement | 8 (17) |
| During food reintroduction | 3 (7) |
| Between months 6-9 (continued weight loss/weight maintenance) | 2 (4) |
| Use of NHS mental health services | |
| Previous NHS mental health services referral | 9 (20) |
| Triages to other NHS mental health service during the programme | 7 (15) |

N (%), Median (IQR)

| Supplementary Table 6: Potentially diet related adverse events in the immediate and delayed intervention groups | | | | | | | | |
| --- | --- | --- | --- | --- | --- | --- | --- | --- |
| Potentially diet related adverse events | Immediate Intervention during initial 3 months of TDR (n=29) | | | | Delayed intervention during 3 months of normal diet (n=16) | | | |
|  | None | Grade 1 | Grade 2 | Grade 3 | None | Grade 1 | Grade 2 | Grade 3 |
| Alopecia | 27 (93.1) | 2 (6.9) | - | - | 15 (93.8) | 1 (6.2) | - | - |
| Constipation | 13 (44.8) | 16 (55.2) | - | - | 13 (81.2) | 3 (18.8) | - | - |
| Diarrhoea | 19 (65.5) | 8 (27.6) | 2 (6.9) | - | 12 (75) | 3 (18.8) | 1 (6.2) | - |
| Dizziness | 15 (51.7) | 14 (48.3) | - | - | 11 (68.8) | 5 (31.2) | - | - |
| Fatigue | 19 (65.5) | 9 (31) | - | 1 (3.4) | 8 (50) | 7 (43.8) | 1 (6.2) | - |
| Headache | 10 (34.5) | 19 (65.5) | - | - | 11 (68.8) | 4 (25) | 1 (6.2) | - |
| Indigestion | 23 (79.3) | 6 (20.7) | - | - | 12 (75) | 4 (25) | - | - |
| Other | 20 (69) | 9 (31) | - | - | 12 (75) | 4 (25) | - | - |

N (%)

Grades scored using common terminology criteria for adverse events [CTCAE v5](1)

References:

1. National Cancer Institute. Common Terminology Criteria for Adverse Events (CTCAE) Common Terminology Criteria for Adverse Events (CTCAE) v5.0. 2017. Available from: <https://www.meddra.org/>
